# Latent Class Analysis of Suicide Methods and Associated Background Characteristics: A Forensic Epidemiological Study in Osaka

**DOI:** 10.31662/jmaj.2025-0376

**Published:** 2026-02-20

**Authors:** Ryu Murakami, Daigo Morioka, Kenko Fukui, Atsushi Hiraide, Hisanaga Kuroki

**Affiliations:** 1 Faculty of Emergency Medical Science, School of Health Science and Medical Care, Meiji University of Integrative Medicine, Nantan, Japan; 2 Graduate School of Risk and Crisis Management, Chiba Institute of Science, Choshi, Japan; 3 Osaka Prefectural Medical Examiner’s Office, Osaka, Japan

**Keywords:** cluster analysis, epidemiology, forensic medicine, Japan, latent class analysis, psychiatric history, suicide methods, suicide attempt

## Abstract

**Introduction:**

Suicide remains a major public health issue in Japan, where the suicide mortality rate is high across all age groups. While existing national statistics provide limited insight into the individual backgrounds of those who die by suicide, detailed forensic data offer an opportunity to explore the characteristics associated with different methods of suicide.

**Methods:**

We conducted a retrospective study using anonymized data extracted from police documents submitted to the Osaka Medical Examiner’s Office for medicolegal investigation. Among 1,129 individuals who died by suicide in 2017 and 2019, 669 cases with complete data were analyzed. Latent class analysis (LCA) was used to classify individuals based on variables, including suicide method, age group, sex, occupation, psychiatric consultation history, suicide attempt history, and living arrangement.

**Results:**

Latent class analysis identified three distinct classes. Class 1 (38.1%) consisted mainly of middle-aged unemployed females with a history of psychiatric consultation, living with others, and frequently jumping from heights. Class 2 (35.1%) was characterized by elderly unemployed males with no history of psychiatric consultation and no suicide attempt history, predominantly using hanging. Class 3 (26.8%) comprised younger employed males with no history of psychiatric consultation and no suicide attempt history, also showing a high proportion of hanging and jumping from heights, and poisoning.

**Conclusions:**

The use of LCA revealed distinct subgroups of suicide deaths characterized by background factors and method choice. These findings may aid in identifying vulnerable populations and inform the development of targeted suicide prevention strategies in Japan. More broadly, our results highlight the value of combining medicolegal information with data-driven classification methods to better understand suicide in other settings.

## Introduction

Suicide is a significant global public health issue that must be addressed at an international level ^(1)^. Japan has one of the highest suicide mortality rates among developed nations, and suicide is the leading cause of death among young people ^(2)^. Furthermore, the country faces major demographic challenges, including a declining birthrate and an aging population, which underscore the urgency of preventing suicide across all age groups ^(3), (4), (5)^.

Our research team has conducted several epidemiological studies on suicide from a forensic medicine perspective ^(6), (7), (8), (9)^. In Japan, few studies have comprehensively examined the medical backgrounds and specific methods of suicide deaths, indicating that foundational research is still in progress.

In Japan, deaths by suicide are classified as “unnatural deaths” ^(10), (11)^. When a body is reported under this category, it is initially investigated by the police or a physician designated by the police to determine whether a crime may have been involved. If criminal involvement is ruled out, further investigations are conducted by a medical professional to determine the cause of death. In certain regions, Japan has implemented a medical examiner system to investigate unnatural deaths, including suicides, from a public health perspective ^(12)^. These medical examiner offices conduct forensic investigations and collect detailed information such as demographic variables, medical records, and suicide methods to clarify the circumstances surrounding death.

There are notable differences between the methods of suicide observed in Japan and those reported in other countries. For example, strict firearm control and regulated access to toxic substances in Japan limit firearm and pesticide suicides ^(13), (14)^.

However, it is difficult to statistically examine the relationship between suicide methods and the demographic or clinical characteristics of individuals―such as sex, age, or history of medical care―using Japan’s primary governmental suicide statistics, including the Vital Statistics and the Suicide Statistics provided by the government ^(2)^. As a result, it is difficult to address the question, “What types of background characteristics are associated with individuals who died by suicide?” and existing reports provide only limited insight into this issue.

This study aimed to investigate the associations between suicide methods and individual characteristics such as age, sex, history of medical care, and other background factors among individuals who died by suicide. Additionally, we sought to classify individuals into subgroups with similar features through clustering analysis, in order to identify distinct typologies of those who died by suicide. This approach is expected to clarify risk profiles that are not visible in standard vital statistics by integrating medicolegal data with latent class analysis (LCA). Such a forensic-public health research framework is not only important for suicide prevention in Japan but could also inform the development of a structured suicide surveillance system internationally.

## Materials and Methods

### Study design

This is a retrospective analysis based on data extracted from official documents submitted by the police to the medical examiner when requesting postmortem investigations of unnatural deaths. These documents included detailed information on individuals who died by suicide (hereafter referred to as “Target Documents”).

### Characteristics of the data

The study was conducted in Osaka City, which operates under Japan’s medical examiner system. In cases where the police determined that the likelihood of criminal involvement was low, the medical examiner conducted a postmortem investigation―including autopsy―to clarify the cause of death. The data used in this study were obtained from the Target Documents, which contain detailed information about suicides identified through police investigations. These documents include information gathered from related institutions such as hospitals and local government offices, including psychiatric consultation history, presence or absence of cohabitants, methods of suicide, and history of suicide attempts.

While the cases analyzed primarily involved unnatural deaths that occurred within Osaka City, the medical examiner’s office also handled cases from outside the city upon request from the police or other authorities. Therefore, the dataset includes some cases that occurred outside the city limits.

### Contents of the target documents

The target documents used in this study were pre-investigation reports submitted by the police to the medical examiner prior to postmortem examinations of unnatural deaths. From these, only cases determined to be suicides were extracted. The documents included the following key items: (1) date and time of discovery, (2) sex, (3) age (recorded as actual number), (4) occupation, (5) presence of cohabitants, (6) history of psychiatric consultation, (7) method of suicide, and (8) free-text notes written by the investigating police officer describing the circumstances of death.

All items except for age and the free-text field were standardized as checkbox entries. The authors compiled these data into a database using Microsoft Excel (Microsoft Corporation, USA). The documents sometimes included a presumed date and time of death; however, this information was not consistently available. In several cases, the body was discovered after an unknown interval following death, and the time of death could only be approximately determined based on postmortem findings. As a result, temporal variables such as weekday or time of death were not analyzed in the present study.

### Study population

This study included anonymized data on 1,129 individuals who died by suicide, as recorded in the target documents processed by the Osaka Medical Examiner’s Office in 2017 and 2019. Cases with missing values in either outcome variables or explanatory variables were excluded from the analysis.

### Statistical analysis

To summarize the characteristics of the study population, continuous variables were presented as medians with interquartile ranges (Q1-Q3), and categorical variables were expressed as counts and percentages. To identify associations between suicide methods and demographic or medical characteristics, we conducted LCA, a multivariate statistical method that classifies a population into unobserved subgroups based on shared characteristics. LCA uses a soft clustering approach by estimating the conditional response probabilities (CRPs), which reflect the likelihood of each individual belonging to each latent class ^(15), (16)^.

The variables included in the LCA were as follows:

(1) method of suicide (a. hanging, b. jumping from heights, c. jumping in front of a train, d. drowning, e. poisoning, f. other [sharp instruments or asphyxiation other than hanging]);

(2) sex (a. male, b. female);

(3) age group (a. young [≤39], b. middle-aged [40-59], c. older [≥60]);

(4) occupation (a. unemployed, b. employed, c. student);

(5) living alone/living with others;

(6) psychiatric consultation history (absence/presence);

(7) suicide attempt history (absence/presence).

The threshold for statistical significance was set at p < 0.05 (two-tailed). The optimal number of latent classes was determined based on the minimum values of the Bayesian Information Criterion (BIC) and Akaike Information Criterion (AIC). In cases where BIC and AIC yielded divergent results, the number of classes was selected based on interpretability and consensus among the authors, considering the data characteristics ^(15)^.

Information on sex was extracted based on the male/female checkbox provided in the target documents. This classification reflects biological sex as recorded in official records, and no data on gender identity were available. Age categories were defined according to previous studies ^(6), (7), (17)^: young (≤39 years), middle-aged (40-59 years), and older adults (≥60 years). Individuals who were living with family members, or residing in institutional facilities (e.g., nursing homes or hospitals) with routine staff visits, were considered to have cohabitants. Descriptive statistics were performed to examine the background characteristics of the groups identified through LCA. All statistical analyses were conducted using JMP Pro 18.0.2 (JMP Statistical Discovery LLC, USA).

### Ethical considerations

This study was approved by the Human Research Ethics Committee of Meiji University of Integrative Medicine (Approval No.: 2022-039). Authorization to access and review the relevant documents was obtained from the Osaka Medical Examiner’s Office. Due to privacy protection regulations, the documents used in this study are not publicly accessible. The study was conducted using an opt-out approach, and explanatory documents regarding the opt-out procedure were made available on the website of the first author’s affiliated institution and displayed in designated areas within the institution.

## Results

### Background characteristics of suicide cases

**Figure 1.**
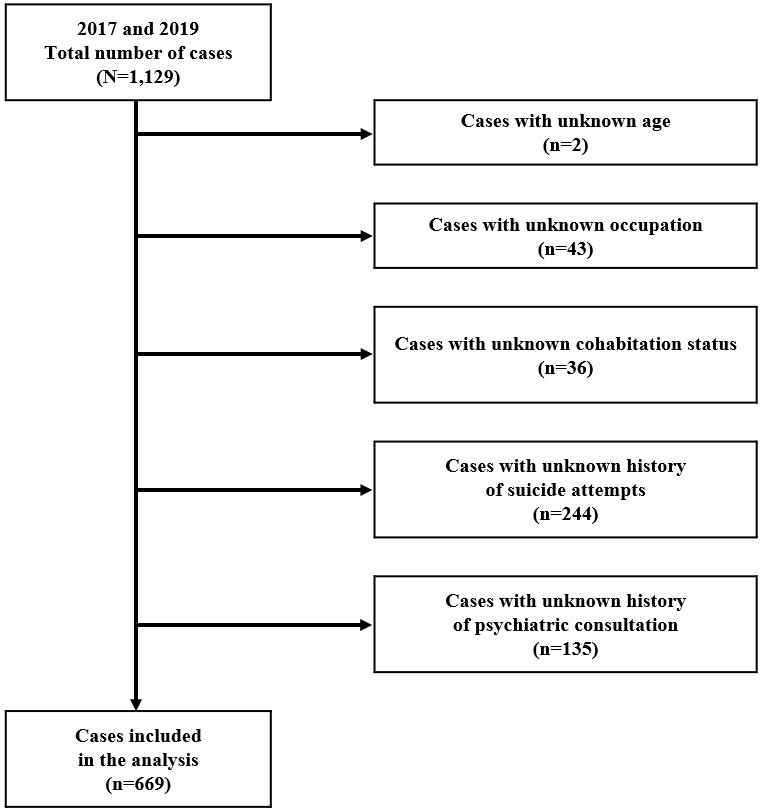
Flowchart of case selection and exclusion of suicide cases with missing data. Among the 1,129 recorded cases, we excluded those with missing values, resulting in 669 cases included in the analysis.

Of 1,129 cases, 669 with complete data were analyzed (Figure 1); their characteristics are in Table 1. Hanging was the most frequently used method of suicide (50.4%). The majority of all cases were male (60.7%), and the median age across both sexes was 52.0 years. Age group distribution was as follows: older (36.0%), middle-aged (35.3%), and young individuals (28.7%).

**Table 1.** Distribution of Demographic and Clinical Variables by Suicide Method.

		Suicide method													
		Total		Hanging		Jumping from heights		Jumping in front of a train		Drowning		Poisoning		Sharp objects etc,	
Variable	n (%)	669	(100.0)	337	(50.4)	212	(31.7)	12	(1.8)	34	(5.1)	54	(8.1)	20	(3.0)
Sex															
	Male	406	(60.7)	222	(65.9)	108	(50.9)	9	(75.0)	22	(64.7)	31	(57.4)	14	(70.0)
	Female	263	(39.3)	115	(34.1)	104	(49.1)	3	(25.0)	12	(35.3)	23	(42.6)	6	(30.0)
Age, Median (Q1-Q3)		52.0	(36.0-68.0)	54.0	(39.5-70.0)	51.0	(35.0-63.0)	44.0	(29.0-82.3)	71.5	(55.5-78.3)	32.5	(23.0-50.0)	66.5	(42.3-71.0)
Age group															
	Young (0-39 yr)	192	(28.7)	84	(24.9)	63	(29.7)	5	(41.7)	2	(5.9)	34	(63.0)	4	(20.0)
	Middle-aged (40-59 yr)	236	(35.3)	117	(34.7)	88	(41.5)	3	(25.0)	8	(23.5)	16	(29.6)	4	(20.0)
	Older (60-99 yr)	241	(36.0)	136	(40.4)	61	(28.8)	4	(33.3)	24	(70.6)	4	(7.4)	12	(60.0)
Occupation															
	Unemployed	423	(63.2)	210	(62.3)	140	(66.0)	7	(58.3)	24	(70.6)	30	(55.6)	12	(60.0)
	Employed	216	(32.3)	118	(35.0)	56	(26.4)	5	(41.7)	10	(29.4)	20	(37.0)	7	(35.0)
	Student	30	(4.5)	9	(2.7)	16	(7.5)	0	(0.0)	0	(0.0)	4	(7.4)	1	(5.0)
Living															
	Living alone	266	(39.8)	133	(39.5)	74	(34.9)	4	(33.3)	12	(35.3)	31	(57.4)	12	(60.0)
	Living with others	403	(60.2)	204	(60.5)	138	(65.1)	8	(66.7)	22	(64.7)	23	(42.6)	8	(40.0)
Suicide attempt history: Presence		179	(26.8)	86	(25.5)	58	(27.4)	1	(8.3)	10	(29.4)	21	(38.9)	3	(15.0)
Psychiatric consultation history: Presence		391	(58.4)	186	(55.2)	142	(67.0)	6	(50.0)	15	(44.1)	34	(63.0)	8	(40.0)

Q1-Q3: 25 to 75 percentile.

Regarding occupational status, unemployed individuals accounted for the largest proportion (63.2%). The majority of individuals lived with others (60.2%). Most had no history of suicide attempts (73.2%), while 58.4% had a history of psychiatric consultation.

When characteristics were examined by suicide method, the proportion of males was highest among those who died by hanging (65.9%). Among individuals who died by poisoning, the median age was the lowest (32.5 years), whereas for drowning, the median age was the highest (71.5 years). In the drowning group, a high proportion were unemployed (70.6%). A high percentage of individuals who died by jumping from heights (67.0%) and poisoning (63.0%) had a history of psychiatric consultation.

#### LCA of suicide methods and background factors

In LCA, lower values of information criteria such as the BIC and AIC indicate better model fit ^(15)^. In our analysis of suicide methods and background factors (Table 2), the lowest BIC was observed in the three-class model (BIC = 7,504.1), while the lowest AIC was observed in the seven-class model (AIC = 7,234.9). Given that BIC is generally considered a more reliable indicator of model fit in LCA ^(15), (18)^, and that the three-class model offered better interpretability based on the characteristics of the data and the practical utility of identifying preventive approaches tailored to each group, we selected the three-class model.

**Table 2.** Model Fit Comparison Based on Information Criteria (AIC and BIC).

Number of Classes	(−1) × Log-Likelihood	BIC		AIC	
2	3717.8	7611.2		7489.5	
3	3618.7	7504.1	*	7319.3	
4	3581.7	7521.1		7273.3	
5	3551.4	7551.7		7240.8	
6	3537.3	7614.5		7240.5	
7	3520.5	7672.0		7234.9	*
8	3508.9	7740.0		7239.8	

AIC: Akaike Information Criterion; BIC: Bayesian Information Criterion. *Indicates the model with the minimum information criterion

Each individual in the dataset was assigned to one of the three latent classes (Table 3). The most prominent CRPs in each class were as follows:

Class 1 (38.1%): Female (64.3%), middle-aged group (53.8%), unemployed (74.4%), jumping from heights (44.0%), living with others (65.2%), history of suicide attempts/presence (53.0%), and history of psychiatric consultation/presence (91.2%).

Class 2 (35.1%): Male (68.5%), older group (84.8%), unemployed (89.4%), hanging (59.7%), living with others (55.7%), history of suicide attempts/absence (87.1%), and history of psychiatric consultation/absence (54.1%).

Class 3 (26.8%): Male (86.1%), young group (63.3%), employed (71.0%), hanging (52.1%), living with others (59.1%), history of suicide attempts/absence (92.3%), and history of psychiatric consultation/absence (71.8%).

**Table 3.** Conditional Response Probabilities and Case Counts for Each Variable by Latent Class.

	Class 1		Class 2		Class 3	
	N*	CRP	N*	CRP	N*	CRP
Variable	251	38.1%	243	35.1%	175	26.8%
Sex						
Male	86	35.7%	165	68.5%	155	86.1%
Female	165	64.3%	78	31.5%	20	13.9%
Age						
Young (0-39 yr)	85	30.4%	0	0.4%	107	63.3%
Middle-aged (40-59 yr)	139	53.8%	29	14.8%	68	35.8%
Older (60-99 yr)	27	15.7%	214	84.8%	0	0.9%
Occupation						
Unemployed	190	74.4%	221	89.4%	12	12.9%
Employed	60	25.1%	22	10.6%	134	71.0%
Student	1	0.6%	0	0.1%	29	16.1%
Methods of Suicide						
Hanging	99	40.5%	147	59.7%	91	52.1%
Jumping from heights	110	44.0%	51	20.8%	51	28.3%
Jumping in front of a train	3	1.1%	4	1.8%	5	2.9%
Drowning	4	1.6%	25	10.9%	5	2.5%
Poisoning	35	12.7%	3	1.2%	16	10.6%
Sharp objects etc,	0	0.2%	13	5.6%	7	3.7%
Living						
Living alone	90	34.9%	108	44.3%	68	40.9%
Living with others	161	65.2%	135	55.7%	107	59.1%
Suicide attempt history						
Absence	103	47.0%	222	87.1%	165	92.3%
Presence	148	53.0%	21	12.9%	10	7.7%
Psychiatric consultation history						
Absence	17	8.8%	132	54.1%	129	71.8%
Presence	234	91.2%	111	45.9%	46	28.2%

CRP: conditional response probability. *Modal assignment.

**Figure 2.**
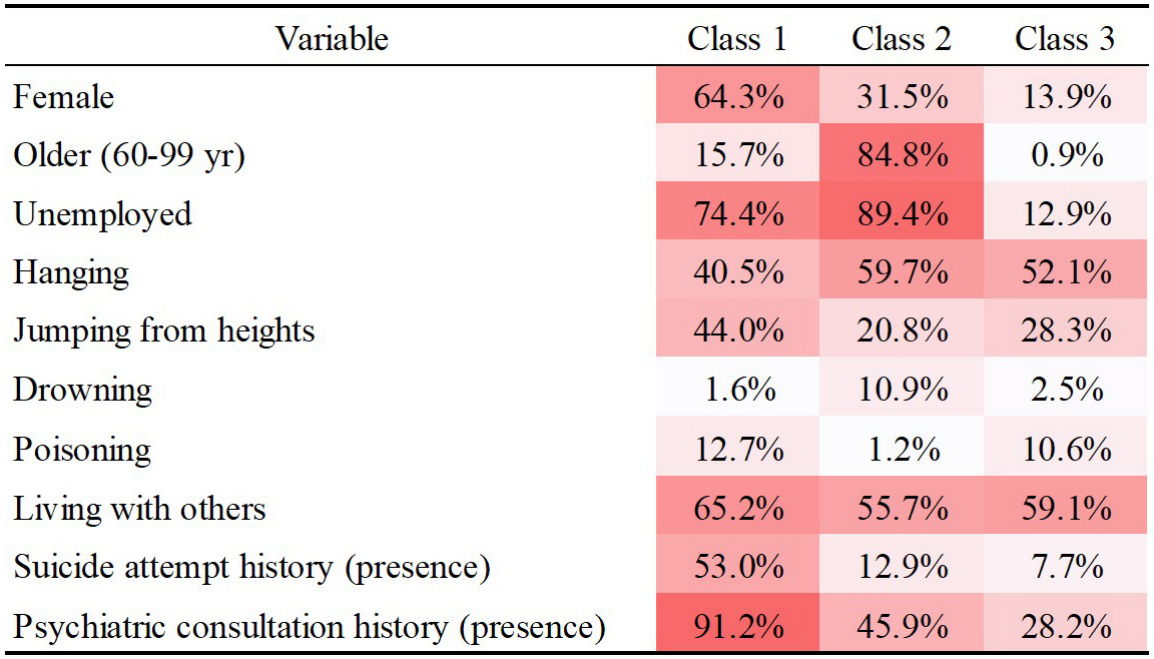
Heatmap of CRPs for selected variables across the three latent classes. Darker shading indicates a higher CRP for that characteristic within the given class. The full CRP matrix, including all variables, is provided in Supplementary Material. CRP: conditional response probability.

Because CRPs are model-based conditional probabilities rather than observed frequencies, small non-zero values may appear even when no cases are assigned to a given class-category combination. Figure 2 provides a visual summary of the CRPs for the key variables that most clearly differentiated the three latent classes. Darker shading indicates a higher CRP, meaning that the characteristic is more typical of that class. The full CRP matrix for all variables is presented in Supplementary Material.

## Discussion

In this study, individuals who died by suicide were classified into three latent classes, and characteristic patterns were identified between suicide methods and factors such as sex and occupational status.

Class 1 was characterized by females, middle-aged individuals, unemployed status, living with others, a history of suicide attempts/presence, psychiatric consultation history/presence, and the use of jumping from heights as the suicide method. These findings suggest a distinctive profile for individuals who chose jumping as their method of suicide. Previous studies have indicated that suicide methods, lethality, and the time course leading to suicide may vary by sex ^(8), (19), (20), (21), (22)^. It has also been reported that the prevalence of self-injurious behavior and suicide attempts is significantly more serious among females than males ^(23)^. In our findings, the presence of cohabitants, history of suicide attempts, and psychiatric consultation history may be interrelated. We hypothesize that non-lethal self-injury occurring in the presence of cohabitants might prompt an emergency response by those cohabitants, leading to medical intervention and subsequent psychiatric referral by emergency medical personnel. Furthermore, the high CRP for psychiatric consultation in this class may imply a link between psychiatric symptoms or disorders and the choice of jumping as a suicide method. This interpretation is consistent with our previous research ^(8)^, highlighting the need for further investigation focused on this perspective.

In addition, poisoning showed the third-highest CRP (12.7%) in this class, following hanging and jumping from heights. This may reflect behaviors related to deliberate drug overdose as a form of self-harm or part of complex self-injurious behavior ^(24), (25), (26)^. However, the present study did not assess the types or quantities of substances involved in poisoning cases, nor whether multiple methods of self-harm were used simultaneously. Further research specifically addressing these aspects is warranted.

Class 2 was characterized by male sex, older age, unemployed status, hanging as the primary suicide method, living with others, history of suicide attempts/absence, and psychiatric consultation history/absence. These findings highlight a distinct profile of older males who chose hanging as their method of suicide. Previous studies have suggested that individuals who have not accessed mental health services and have no history of suicide attempts may be more likely to use highly lethal or violent methods ^(27)^.

Compared to Class 1, Class 2 was notable in that individuals often had no psychiatric consultation history and no history of suicide attempts―even when cohabitants were present. The CRP for drowning (10.9%) was also relatively higher in this class compared to the same method in other classes.

A previous study investigating the relationship between the increasing number of high-rise buildings and suicides by jumping among older adults noted that preventive measures, such as installing safety fences, can be effective in reducing suicide rates ^(28)^. In the context of our findings, it is possible that drowning, compared to methods like jumping from heights or poisoning, may be more physically feasible or require less preparation―particularly for older adults. This could also relate to conditions more prevalent in older populations, such as dementia or insomnia, or to the effects of treatment for these conditions.

However, our study did not examine individual diagnoses―either physical or psychiatric―or the specific medications or treatments being administered to the decedents. Future studies focusing on these aspects are warranted to better understand the context behind method selection among older adults.

Class 3 was characterized by male sex, younger age, employed status, hanging as the primary suicide method, living with others, and a history of suicide attempts/absence and psychiatric consultation/absence. These results highlight a distinct profile of younger, employed males who died by suicide using hanging.

The association between sex and suicide method observed in this class supports findings from the analyses of Classes 1 and 2, suggesting that the high lethality of hanging may be a contributing factor in method selection ^(29)^. Furthermore, the CRP for jumping from heights in Class 3 was 28.3%, higher than that in Class 2 (20.8%), which was characterized by older males. This difference suggests a potential relationship between the choice of jumping as a method and variables such as age or employment status. For instance, younger, employed individuals may have easier access to high-rise buildings (e.g., workplaces) or greater physical ability to overcome barriers such as fences compared to older adults.

The CRP for poisoning (10.6%) was the second highest among the three classes, following Class 1. This finding may reflect the current context in Japan, where drug use is increasing among young people ^(24), (26)^, and where access to potentially lethal over-the-counter medications may be more feasible for younger individuals than for older adults (Class 2), possibly due to their greater ability to gather information online or through social media ^(30)^.

However, the limitations discussed in relation to Class 1’s findings on poisoning also apply here. Specifically, this study did not investigate the means of acquiring the substances used, nor did it examine the types or quantities of drugs involved. Further research is needed to address these aspects and better understand the factors influencing poisoning-related suicides among younger populations.

These findings demonstrate that each latent class is characterized by distinct combinations of background factors and that multiple variables may interact in complex and latent ways among individuals who died by suicide. From a suicide prevention perspective, the three-class categorization identified in this study suggests distinct and complementary directions for intervention and education.

For Class 1, improving the continuity of psychiatric care and community follow-up after non-lethal self-harm is essential. Strengthening linkages between emergency medical services, psychiatric departments, and community support networks may help prevent recurrence. In addition, mental health literacy and support programs ^(31)^ directed at cohabitants may be beneficial, as cohabitants are often the first to recognize warning signs and initiate help-seeking.

For Class 2, outreach and education programs aimed at reducing stigma and encouraging help-seeking behavior are critical. Collaboration with primary care providers, community centers, and other welfare organizations could facilitate earlier detection and intervention of distress among socially isolated elderly individuals ^(32)^.

For Class 3, workplace and media-based mental health promotion may be more effective ^(33)^. Initiatives that normalize discussion of psychological stress and provide accessible digital counseling resources could lower barriers to seeking help.

Visualizing these latent profiles can also contribute to public awareness by illustrating that suicide risk is heterogeneous and that prevention strategies must be multifaceted rather than uniform.

Spatial and social context may influence both access to specific suicide methods and opportunities for intervention. Even within urban settings, environmental conditions are not uniform: areas with high-rise office districts and large daytime working populations differ from primarily residential districts, and locations with frequent rail traffic differ from areas without such infrastructure. These environments may shape both the feasibility of particular methods (for example, jumping from heights or in front of a train) and the likelihood that distress is witnessed or responded to by others. Because the present analysis was restricted to Osaka City, it reflects one specific urban context. Applying the same forensic-epidemiological approach to other regions, including non-urban areas, will be essential to clarify how these contextual factors vary across Japan. Developing a nationwide framework that systematically collects postmortem information on individuals who died by suicide would greatly enhance such comparisons.

Since this study focused on individuals who ultimately died by suicide, analyzing the relationships between their suicide methods and background characteristics can help identify vulnerable populations. Such identification is crucial for informing policy decisions related to suicide prevention. Ongoing and continuous investigation is therefore essential.

Although the present study analyzed data from a limited period (2017 and 2019), it represents one of the few attempts to apply an epidemiological framework to medicolegal data on suicide deaths. The Target Documents used here were originally created by police and medical examiners for administrative and investigative purposes, not for research, yet they contain rich information that can reveal important public health insights when analyzed systematically. Looking ahead, the integration of both fatal and non-fatal suicidal behaviors into a standardized registry, similar to the Utstein-style reporting system for out-of-hospital cardiac arrest ^(34)^, which established internationally comparable standards for reporting critical events, could facilitate cross-regional comparisons and promote evidence-based suicide prevention from a combined forensic and public health perspective.

This study has several limitations.

First, this study included only individuals who died by suicide and did not capture non-fatal suicide attempts.

Second, all cases were drawn from Osaka City, which is covered by Japan’s medical examiner system. The findings may therefore not be directly generalizable to other regions in Japan or to other countries.

Third, the analysis relied on document-based information, and some relevant factors may have been unknown or not recorded at the time of submission to the medical examiner’s office.

Fourth, temporal information was incomplete. In some cases, the presumed date and time of death were documented, but in others―particularly when the body was found several days later―the time of death could only be approximated. We therefore did not analyze variables such as weekday or time of death. Temporal patterns remain important and should be addressed in future studies.

Finally, approximately 40% of identified cases were excluded from the LCA because of missing information, mainly regarding psychiatric consultation history and suicide attempt history. This exclusion may have introduced selection bias toward cases with more detailed investigative information, and the identified class structure may not fully represent all suicide deaths during the study period.

### Conclusions

This study examined the relationship between suicide methods and background characteristics of individuals who died by suicide, using data obtained from a forensic medicine perspective. Through LCA, we identified three distinct subgroups with similar background profiles.

Suicide is a multifactorial phenomenon that arises from the complex interplay of various elements; thus, the findings of this study do not imply that the presence of specific factors directly causes suicide. However, the use of analytical methods such as LCA to identify vulnerable groups may provide a meaningful basis for developing more robust evidence to support preventive interventions.

It is essential to further enhance interdisciplinary collaboration among forensic medicine, psychiatry, neuroscience, and other related fields, and to promote active investigation not only of individuals who attempt suicide but also of those who have died by suicide. Broader perspectives will contribute to a more comprehensive understanding of suicide and inform effective prevention strategies.

## Article Information

### Acknowledgments

The authors express their gratitude to the Osaka Prefectural Medical Examiner’s Office for providing the data used in this study.

### Authors Contributions

Conceptualization, Investigation, Writing - Original Draft, Project administration: Ryu Murakami. Conceptualization, Methodology, Validation, Investigation: Daigo Morioka.

Methodology, Validation, Investigation: Kenko Fukui. Methodology, Supervision, Writing - Review & Editing: Atsushi Hiraide. Resources, Investigation, Supervision: Hisanaga Kuroki. Read and agreed to the published version of the manuscript: all authors.

### IRB Approval Code and Name of the Institution

The study was conducted with the approval of the Human Ethics Review Committee of Meiji University of Integrative Medicine (Approval No.: 2022-039).

### Conflicts of Interest

None

### Patient Consent

This study was conducted in accordance with an opt-out procedure. The opt-out document, prepared in compliance with Japan’s ethical guidelines, allows the families of the deceased to freely request exclusion from the study if they wish. This document is available on the website of the research institution to which the first author belongs, and printed versions are posted and available at designated locations within the author’s affiliated institution.

### Data Availability

The information on individuals who died by suicide used in this study is based on documents stored at the Osaka Prefectural Medical Examiner’s Office. Therefore, this information is not publicly available. To conduct this study, materials were processed to ensure that individuals could not be identified. The statistical analyses in this study were conducted on information terminals that were disconnected from the internet and external networks. The data used were stored on encrypted storage media and strictly managed within the research institution to which the first author is affiliated.

## Supplement

Supplementary Material Full heatmap of CRPs for all variables included in the latent class analysis.
Darker shading indicates a higher CRP for that characteristic within the given class.
This comprehensive figure complements Figure 2, and allows detailed inspection of the full latent class structure.
CRP: conditional response probability.
