# Supplementary material for: Latent Class Analysis of Suicide Methods and Associated Background Characteristics: A Forensic Epidemiological Study in Osaka
Source: JMA J. 2026 Feb 20;9(2):476–85. doi: 10.31662/jmaj.2025-0376 (PMC13058713; doi:10.31662/jmaj.2025-0376)
Supplement: Supplementary Material — Full heatmap of CRPs for all variables included in the latent class analysis. Darker shading indicates a higher CRP for that characteristic within the given class. This comprehensive figure complements Figure 2, and allows detailed inspection of the full latent class structure. CRP: conditional response probability. [file 2433-3298-9-2_0476-s001.pdf]

Variable	Class 1	Class 2	Class 3
Male	35.7%	68.5%	86.1%
Female	64.3%	31.5%	13.9%
Young (0-39 yr)	30.4%	0.4%	63.3%
Middle-aged (40-59 yr)	53.8%	14.8%	35.8%
Older (60-99 yr)	15.7%	84.8%	0.9%
Unemployed	74.4%	89.4%	12.9%
Employed	25.1%	10.6%	71.0%
Student	0.6%	0.1%	16.1%
Hanging	40.5%	59.7%	52.1%
Jumping from heights	44.0%	20.8%	28.3%
Jumping in front of a train	1.1%	1.8%	2.9%
Drowning	1.6%	10.9%	2.5%
Poisoning	12.7%	1.2%	10.6%
Sharp objects etc,	0.2%	5.6%	3.7%
Living with others	65.2%	55.7%	59.1%
Suicide attempt history (presence)	53.0%	12.9%	7.7%
Psychiatric consultation history (presence)	91.2%	45.9%	28.2%
